# Supplementary material for: An Analysis of Genetic Polymorphisms in 76 Genes Related to the Development of Ovarian Tumors of Different Aggressiveness
Source: Int J Mol Sci. 2024 Oct 10;25(20):10876. doi: 10.3390/ijms252010876 (PMC11507582; doi:10.3390/ijms252010876)
Supplement: Supplementary file 1 [file ijms-25-10876-s001.zip › Szafron LA et al. 2024 - supplement.pdf]

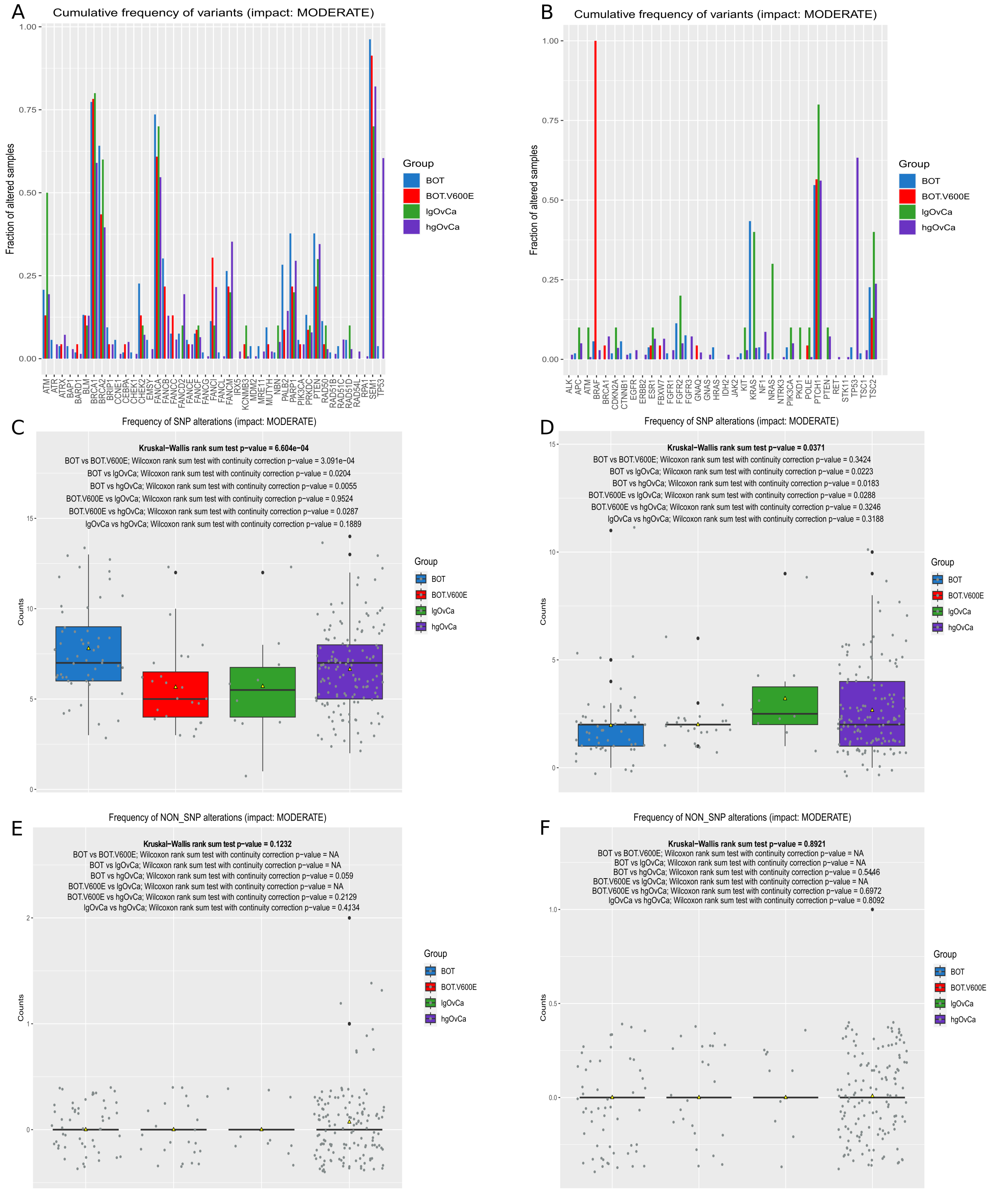

Figure S1. **SNP and non-SNP variants with a moderate impact for both gene panels.** A,C,E: results for the 44-gene panel. B,D,F: results for the hot spot panel. A, B: cumulative frequency of genetic variants (SNPs and non-SNPs combined) with a moderate impact per gene in each tumor group. C-F: box plots demonstrating differences in the numbers of genetic variants between the analyzed groups of tumors, for SNP (C,D) and non-SNP (E,F). Each box plot is additionally supplemented with the Kruskal-Wallis rank sum test (showing whether there is any statistically significant difference in the analyzed sets of variants) and the Wilcoxon rank sum test with continuity correction (the post-hoc test applied to determine which tumor groups differed from each other). NA – result not available, because statistical inference was impossible due to the lack of samples with non-SNP variants in the BOT, BOT.V600E and lgOvCa groups. Group sizes: BOT: N = 53, BOT.V600E: N = 23, lgOvCa: N = 10, hgOvCa: N = 139.

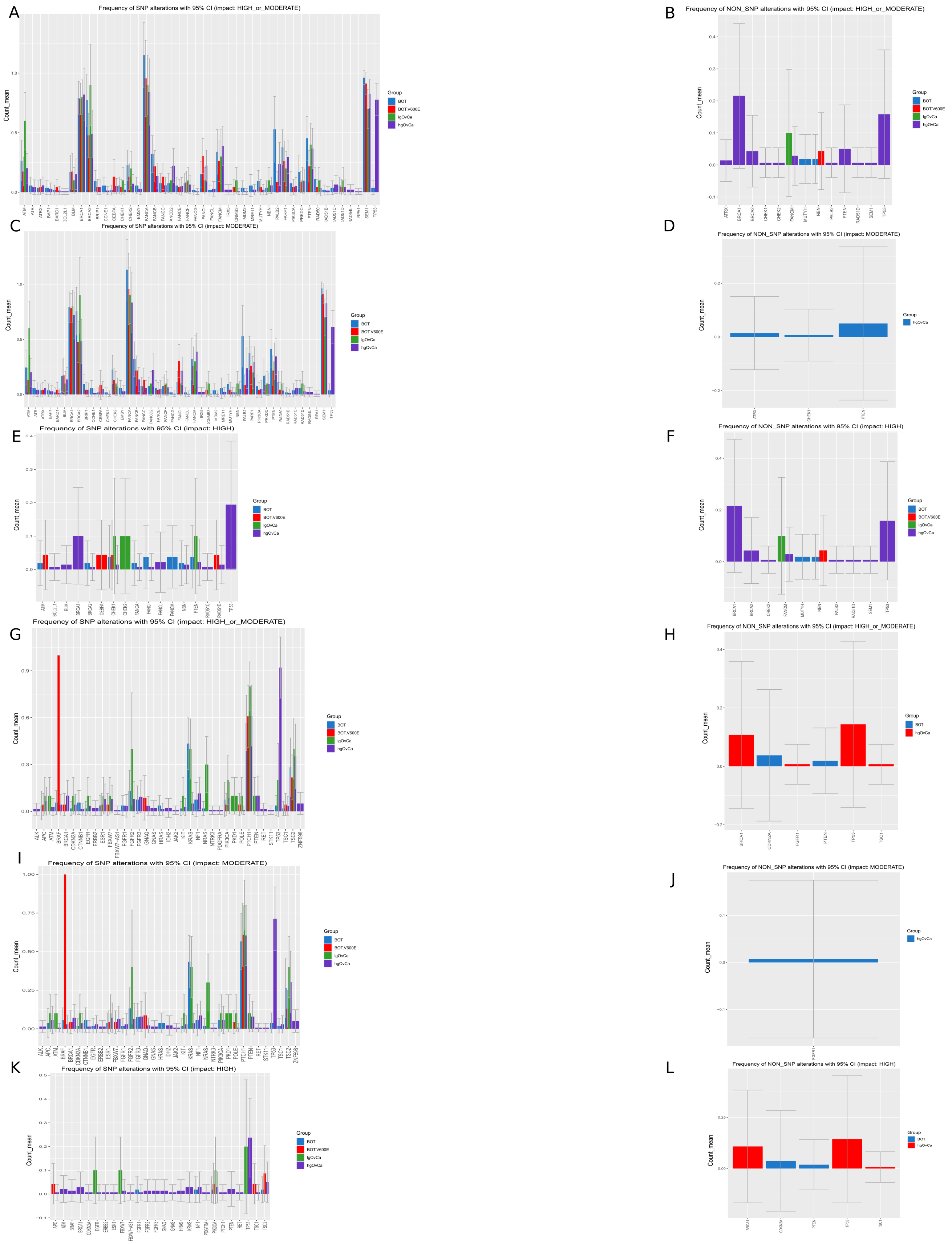

Figure S2. **Mean count of SNP and non-SNP variants per genes (both gene panels).** A-F: 44-gene panel, G-L: hot spot panel. A,C,E,G,I,K: SNP; B,D,F,H,J,L: non-SNP. A,B,G,H: variants with a high or moderate impact; C,D,I,J: variants with a moderate impact; E,F,K,L: variants with a high impact.

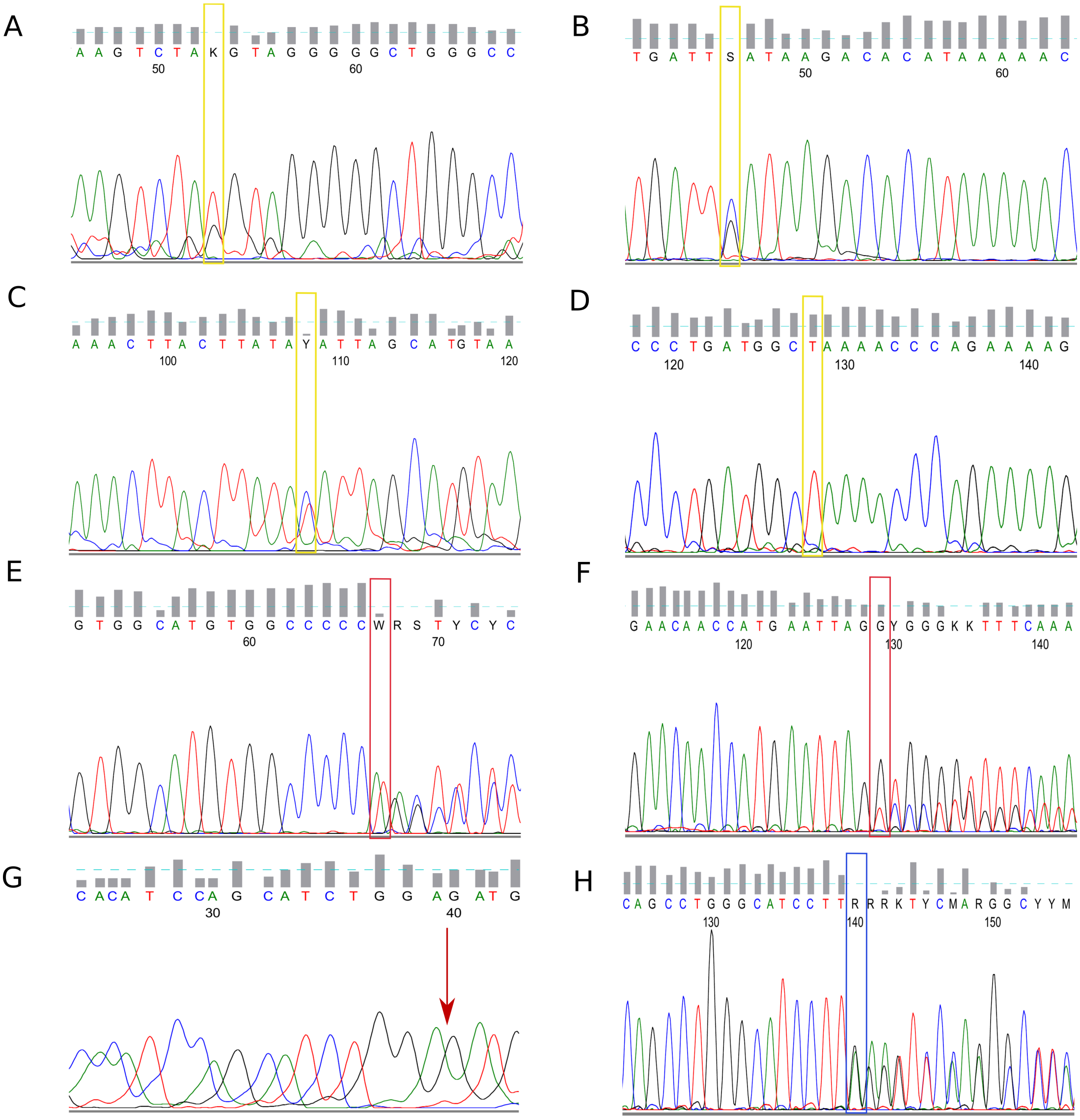

I

Gene name	Variant (SNP/non-SNP)	Group	% of aletered reads
FANCE	chr6:g.35456000T>G	BOT.V600E	38
FANCM	chr14:g.45187852C>G	hgOvCa	40
PRKDC	chr8:g.47779009C>T	hgOvCa	54
FANCI	chr15:g.89295051C>T	hgOvCa	93
MUTYH	chr1:45332673del	BOT	34
BRCA1	chr17:43093093_43093096del	hgOvCa	56
RAD51D	chr17:g.35106436del	hgOvCa	91
TP53	chr17:g.7670658_7670659insA	hgOvCa	53

Figure S3. The r**esults of Sanger sequencing for selected genetic variants** in the following genes: A: *FANCE,* B: *FANCM,* C: *PRKDC,* D: *FANCI,* E: *MUTYH,* F: *BRCA1,* G: *RAD51D* and H: *TP53*. I: A description of each verified variant. Yellow rectangles (A-D) indicate SNP variants, red rectangles (E,F) and a red arrow (G) show deletions, while an insertion is denoted with a blue rectangle. For *FANCI*, and *RAD51D*, altered variants were found in over 90% of reads. Therefore, in both cases, normal sequences were not visible in the chromatograms (D,G).

Table S1: **Clinicopathological characteristics of the cohort of patients with BOTS.**

BRAF.V600E	No	Yes	All_samples
**N**	53	23	76
**Microinvasion_or_implants.No**	36	13	49
**Microinvasion_or_implants.Yes**	17	10	27
**Relapse.No**	46	19	65
**Relapse.Yes**	6	3	9
**Relapse.NA**	1	1	2
**RFS.Min.**	112	293	112
**RFS.1st Qu.**	3668.75	2880.25	3271.25
**RFS.Median**	4677.5	4331.5	4539.5
**RFS.Mean**	4453.83	4033.68	4328.92
**RFS.3rd Qu.**	5488	5515	5523.25
**RFS.Max.**	7205	6190	7205
**RFS.NA**	1	1	2
**Chemotherapy.No**	41	19	60
**Chemotherapy.Yes**	12	4	16
**Primary_tumor.No**	13	7	20
**Primary_tumor.Yes**	39	16	55
**Primary_tumor.NA**	1	0	1
**FIGO.IA-IB**	14	5	19
**FIGO.IC**	14	6	20
**FIGO.IIA-IIIC**	11	5	16
**FIGO.NA**	14	7	21
**Type.other**	15	0	15
**Type.serous**	38	23	61
**Age.Min.**	18	21	18
**Age.1st Qu.**	32	25.5	28
**Age.Median**	44	29	36.5
**Age.Mean**	44.81	32.13	40.97
**Age.3rd Qu.**	55	35.5	52.25
**Age.Max.**	74	76	76
**Frozen_samples**	16	5	21
**FFPE_samples**	37	18	55

RFS – relapse-free survival; Type – histological type; Qu. – quartile; NA – not applicable/missing data

Table S2: **Clinicopathological characteristics of the cohort of patients with OvCa.**

Therapy	PC	PC	TP	TP	All_samples
**TP53.accumulation**	**No**	**Yes**	**No**	**Yes**	
**N**	12	23	42	70	147
**CR.0**	4	9	15	16	44
**CR.1**	8	14	27	54	103
**Relapse.0**	1	1	4	8	14
**Relapse.1**	7	13	23	46	89
**Relapse.NA**	4	9	15	16	44
**Death.0**	0	1	4	14	19
**Death.1**	12	22	38	56	128
**PS.0**	4	14	19	21	58
**PS.1**	8	9	23	49	89
**DFS.Min.**	0	0	0	0	0
**DFS.1st Qu.**	0	0	0	139.75	0
**DFS.Median**	266	127	230.5	363	251
**DFS.Mean**	454.33	384.13	387.55	548.29	469.01
**DFS.3rd Qu.**	548	460.5	484.25	743.5	591.5
**DFS.Max.**	2426	2521	4380	2884	4380
**OS.Min.**	56	81	263	296	56
**OS.1st Qu.**	497.75	587.5	655.75	876.5	683
**OS.Median**	1167	897	853.5	1185.5	1105
**OS.Mean**	1293.17	1186.09	1271.86	1571.21	1402.73
**OS.3rd Qu.**	2046.25	1578	1706.25	1982.25	1864
**OS.Max.**	2742	3750	4500	5630	5630
**Type.other**	0	1	11	15	27
**Type.serous**	12	22	31	55	120
**FIGO.IC-IIC**	0	0	2	2	4
**FIGO.IIIA-IIIB**	1	6	4	5	16
**FIGO.IIIC**	8	15	34	58	115
**FIGO.IV**	3	2	2	5	12
**Grade.lgOvCa**	4	0	4	0	8
**Grade.hgOvCa**	8	23	38	70	139
**RT = 0 cm**	3	5	9	18	35
**RT < 2 cm**	4	6	26	35	71
**RT ≥ 2 cm**	5	12	7	16	40
**RT.NA**	0	0	0	1	1
**Age.Min.**	34	36	20	33	20
**Age.1st Qu.**	44.5	45.5	47.5	47	47
**Age.Median**	49	58	53	53	53
**Age.Mean**	53	55.70	52.60	54.26	53.90
**Age.3rd Qu.**	64.25	64	61.5	61	62.5
**Age.Max.**	68	77	74	84	84
**Frozen_samples**	12	23	42	70	147
**FFPE_samples**	NA	NA	NA	NA	NA

CR – complete remission; PS – platinum sensitivity; DFS – disease-free survival; OS – overall survival; Type – histological type; RT – residual tumor size; PC – platinum/cyclophosphamide; TP – taxane/platinum; NA – not applicable/missing data; Qu. – quartile; 0 – “No”; 1 – “Yes”

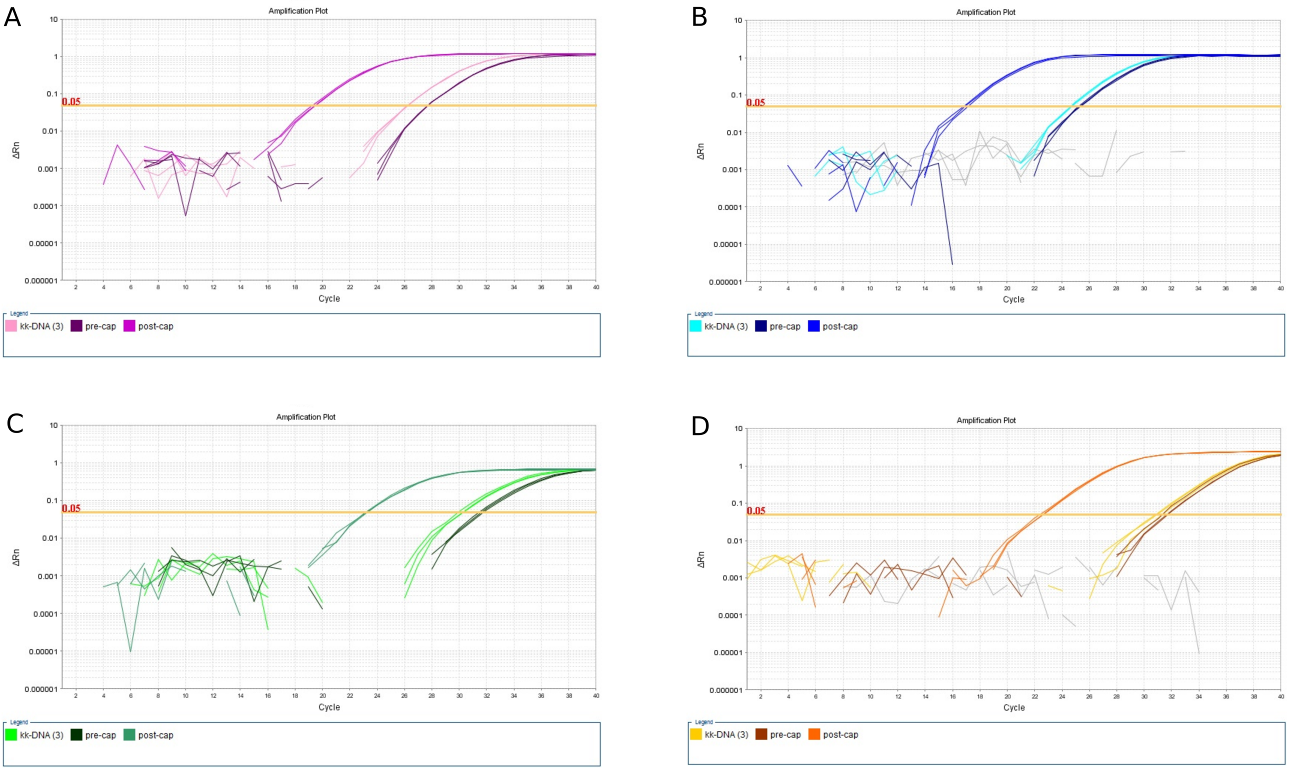

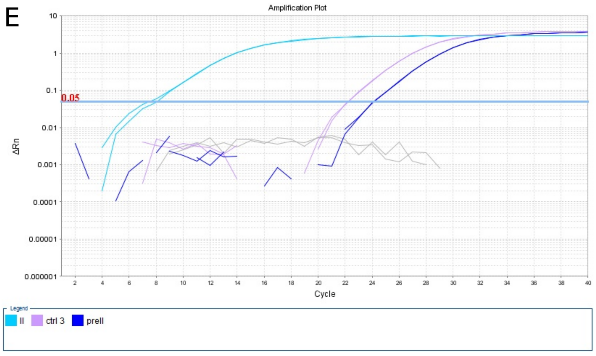

F

Primer name	Sequence (5'---> 3')
qPCR NSC-0237, forward	CGC ATT CCT CAT CCC AGT ATG
qPCR NSC-0237, reverse	AAA GGA CTT GGT GCA GAG TTC AG
qPCR NSC-0247, forward	CCC ACC GCC TTC GAC AT
qPCR NSC-0247, reverse	CCT GCT TAC TGT GGG CTC TTG
qPCR NSC-0268, forward	CTC GCT TAA CCA GAC TCA TCT ACT GT
qPCR NSC-0268, reverse	ACT TGG CTC AGC TGT ATG AAG GT
qPCR NSC-0272, forward	CAG CCC CAG CTC AGG TAC AG
qPCR NSC-0272, reverse	ATG ATG CGA GTG CTG ATG ATG
TP53 exon 4, forward	ACC CAG GTC CAG ATG AAG C
TP53 exon 4, reverse	AGG GGC CGC CGG TGT AGG A

Figure S4. The v**erification of gDNA libraries' enrichment.** A-D: 44-gene panel, E: hot spot panel. In each experiment, the same amount (12-16 ng per well) of normal control gDNA (kkDNA (3)/ctrl 3) and tumor gDNA before (pre-cap/preII), and after (post-cap/II) enrichment was used. The results are presented in the following order: A: NSC-0237, B: NSC-0247, C: NSC-0268, D: NSC-0272, E: in-house designed primers for *TP53* exon 4. Commercial Roche primers are denoted as NSC followed by a number. F: Sequences of each primer pair used for the enrichment verification. This verification was performed for every pool of NGS libraries. However, only exemplary, representative results for each primer pair are displayed here.

Table S3. **PCR and Sanger sequencing primers and conditions used for the verification of selected polymorphisms.**

***MUTYH*, chr1:45332673del (179bp)**						
Primer F:	GCCAAAGGCGATAGAGGCAA					
Primer R:	GATGGCAGGAGGGTAGGAAC					
PCR	95 ºC (5 min)	94 ºC (30 s)	55 ºC (10 s)	72 ºC (15 s)	72 ºC (5 min)	4 ºC (∞)
		40 Cycles				
Sanger	95 ºC (2 min)	96 ºC (10 s)	55 ºC (5 s)	60 ºC (4 min)	4 ºC (∞)	
		35 Cycles				
***BRCA1,* chr17: 43093093_43093096del (239bp)**						
Primer F:	GACTGGCGCTTTGAAACCTT					
Primer R:	AGCACTCTAGGGAAGGCAAAA					
PCR	95 ºC (5 min)	94 ºC (30 s)	55 ºC (10 s)	72 ºC (15 s)	72 ºC (5 min)	4 ºC (∞)
		40 Cycles				
Sanger	95 ºC (2 min)	96 ºC (10 s)	55 ºC (5 s)	60 ºC (4 min)	4 ºC (∞)	
		35 Cycles				
***FANCE,* chr6:g.35456000T>G (170bp)**						
Primer F:	ATTGCTGCGAAGGGATTTGG					
Primer R:	GGGAGTCCCTGTTCTCCTCT					
PCR*	95 ºC (5 min)	94 ºC (30 s)	60 ºC (5 s)	72 ºC (4 s)	72 ºC (5 min)	4 ºC (∞)
		40 Cycles				
Sanger	95 ºC (2 min)	96 ºC (10 s)	60 ºC (5 s)	60 ºC (4 min)	4 ºC (∞)	
		35 Cycles				
***FANCI*, chr15:g.89295051C>T (219bp)**						
Primer F:	CCAAAGCCACCAAGAAAGCC					
Primer R:	ACACCACAGCCAAGTGGAAT					
PCR	95 ºC (5 min)	94 ºC (30 s)	60 ºC (11 s)	72 ºC (30 s)	72 ºC (5 min)	4 ºC (∞)
		40 Cycles				
Sanger	95 ºC (2 min)	96 ºC (10 s)	60 ºC (5 s)	60 ºC (4 min)	4 ºC (∞)	
		35 Cycles				
***FANCM*, chr14:g.45187852C>G (154bp)**						
Primer F:	TCTTTGCGTAGTCCAATGATGA					
Primer R:	ATGGAGGCTTCACTAACAGGAA					
PCR	95 ºC (5 min)	94 ºC (30 s)	60 ºC (10 s)	72 ºC (20 s)	72 ºC (5 min)	4 ºC (∞)
		40 Cycles				
Sanger	95 ºC (2 min)	96 ºC (10 s)	60 ºC (5 s)	60 ºC (4 min)	4 ºC (∞)	
		35 Cycles				
***PRKDC*, chr8:g.47779009C>T (178bp)**						
Primer F:	CTTGATGATCTCTCTGAGGCAA					
Primer R:	CCGCCGTGTGAATATAAAGATTGG					
PCR	95 ºC (5 min)	94 ºC (30 s)	60 ºC (11 s)	72 ºC (30 s)	72 ºC (5 min)	4 ºC (∞)
		40 Cycles				
Sanger	95 ºC (2 min)	96 ºC (10 s)	60 ºC (5 s)	60 ºC (4 min)	4 ºC (∞)	
		35 Cycles				

***RAD51D*, chr17:g.35106436del (131bp)**						
Primer F:	GCAGAACAGCAGGCTCACC					
Primer R:	CTTTTCCACCGCTTCAGGCA					
PCR	95 ºC (5 min)	94 ºC (30 s)	60 ºC (10 s)	72 ºC (30 s)	72 ºC (5 min)	4 ºC (∞)
		40 Cycles				
Sanger	95 ºC (2 min)	96 ºC (10 s)	60 ºC (5 s)	60 ºC (4 min)	4 ºC (∞)	
		35 Cycles				
***TP53*, chr17:g.7670658_7670659insA (246bp)**						
Primer F:	GCTGGGACCCAATGAGATGG					
Primer R:	CTCTGTTGCTGCAGATCCGT					
PCR	95 ºC (5 min)	94 ºC (30 s)	60 ºC (11 s)	72 ºC (30 s)	72 ºC (5 min)	4 ºC (∞)
		40 Cycles				
Sanger	95 ºC (2 min)	96 ºC (10 s)	60 ºC (5 s)	60 ºC (4 min)	4 ºC (∞)	
		35 Cycles				

Emboldened are: the names of genes, analyzed polymorphisms and the lengths of PCR products. All PCRs were performed in the gradient of annealing temperatures ranging from 55 to 65 ºC. In the table, the most optimal annealing temperatures are shown. An asterisk (*) indicates the use of the Phusion Green polymerase instead of the AmpliTaq Gold polymerase. F: forward primer; R: reverse primer.

Table S4. **Western blot conditions.**

Protein	Membrane type	Transfer on membrane	Primary Ab conc.	Incubation with primary Ab
NBN	Nitrocellulose 0.2 μm	Overnight (27 mA)	1:900	3 h (RT)
CHEK2	Nitrocellulose 0.2 μm	Overnight (27 mA)	1:650	3 h (RT)
CHEK1	Nitrocellulose 0.2 μm	1 h (300 mA)	1:2000	Overnight (4 °C)
FANCI	Nitrocellulose 0.2 μm	1 h 15 min (300 mA)	1:1500	Overnight (4 °C)
FANCD2	Nitrocellulose 0.2 μm	1 h 15 min (300 mA)	1:750	Overnight (4 °C)
TP53	PVDF 0.2 μm	1 h (300 mA)	1:250	Overnight (4 °C)

For all cases, except TP53, the secondary HRP-conjugated Ab was used in the final concentration of 1:15,000. For TP53, the secondary HRP-conjugated Ab was used in the final concentration of 1:10,000. RT – room temperature.
